# Family structure and family satisfaction as factors of adolescents’ autonomous motivation, cheating, and academic achievement

**DOI:** 10.1371/journal.pone.0357562

**Published:** 2026-09-16

**Authors:** Tamara O. Gordeeva, Elena V. Dvorskaya, Oleg A. Sychev

**Affiliations:** 1 Department of Psychology, Lomonosov Moscow State University, Moscow, Russia; 2 Federal Scientific Center for Psychological and Interdisciplinary Research, Moscow, Russia; Amrita Vishwa Vidyapeetham - Amritapuri Campus, INDIA

## Abstract

There is considerable evidence showing that children growing up in two-parent intact families are more academically successful than children from other family structures. However, little is known about the psychological mechanisms underlying the relationship between family structure and academic achievement. Therefore, the aim of this study was to examine the role of satisfaction with family relationships as a mediator of the associations of family structure with academic motivation, attitude toward school, and academic achievement. Using a representative sample of Russian adolescents in grades 5–9 (N = 2,402; M_age_ = 12.99, SD = 1.54; 51% girls, 49% boys), we analyzed indicators of academic motivation, homework motivation, persistence, academic achievement, and satisfaction with family relationships among adolescents from three characteristic types of family: intact families (N = 1,757), single-mother families (N = 465), and stepfather families (N = 180). The results show that adolescents from single-mother and stepfather families form a homogeneous group that differs from children from intact families in terms of satisfaction with family relationships, autonomous academic motivation, persistence, attitude toward school, homework cheating and academic achievement, all of which are higher among adolescents from intact families. Furthermore, compared to adolescents from intact families, adolescents from single-mother and stepfather families exhibit greater amotivation, are prone to cheating on homework, and have a less positive attitude toward school. Structural modeling reveals a system of family and motivational factors associated with academic attainment. It is primarily shown that satisfaction with family is a partial mediator of the associations of family structure with autonomous motivation, persistence, and academic achievement. The practical implications of the study are that satisfaction with family relationships can mitigate the risks of adverse development and low achievement in children growing up in single-mother and stepfather families. The results highlight the importance of psychological work with parents of children from single-mother and stepfather families.

## Introduction

In most countries worldwide, the proportion of non-intact families has been increasing, driven both by a rise in the number of women who do not enter marriage and by growing divorce rates [1]. A similar trend is observed in Russia: over the past two decades, the share of non-intact families has increased by 1.8 times and reached 39% in 2022, with single-mother families accounting for 81% of this group [2]. Previous research suggests that the growth of non-intact families may be associated with increasing individualism in society, greater tolerance toward nonmarital relationships and single motherhood, and changes in women’s social roles [3,4]. Moreover, most studies indicate that adolescents from non-intact families demonstrate lower academic achievement than adolescents from intact families [5–9]. However, the psychological factors that may account for this association remain insufficiently understood [10]. The present study addresses this gap by examining satisfaction with family relationships, academic motivation, and persistence as mediators of the association between family structure and adolescents’ academic achievement.

### Associations between family structure and adolescents’ academic achievement

Academic achievement is a key indicator of successful development in children and adolescents, which is accepted as a basic benchmark in schools, predicting future educational attainment and occupational prestige [11]. A substantial body of research indicates that adolescents growing up in non-intact families demonstrate lower academic achievement, including school performance and results on subject-specific standardized tests [5–9]. Compared with adolescents from intact families, they are more likely to disengage from schooling, repeat grades, and drop out of school before completing secondary education [7,12], report lower educational plans and aspirations [13]. Research also shows that adults who were raised in non-intact families during childhood demonstrate lower levels of educational attainment compared with those who experienced living with two biological parents [12,14,15].

Analyses based on data from the large-scale international PISA study, which includes representative samples of 15-year-old adolescents from 28 countries worldwide, show that adolescents growing up in two-parent families demonstrate higher math, science, and reading achievement than those growing up in single-parent families [1]. At the same time, in some countries the difference in academic achievement between adolescents growing up in single-parent and two-parent families is substantial (e.g., Belgium, the Netherlands, and the United States), whereas in others (e.g., Mexico and Portugal) it is almost absent. This cross-national variation is associated with differences in national policies toward families of different types, in particular with financial support provided to single parents [1]. From our perspective, another factor that may account for variation in the association between family structure and academic achievement is adolescents’ satisfaction with their family and family relationships, which, in turn, may be related to the level of individualism–collectivism in these countries. In such contexts, the association between the absence of one parent and adolescents’ academic achievement may be attenuated by frequent interaction and support from other relatives and members of the extended family living nearby.

### Differences among non-intact family structures and adolescents’ academic achievements

Researchers currently identify a wide range of typologies of non-intact families [16], which poses a serious challenge for research on family structure. A limitation of many studies is that different types of non-intact families are often combined in the analysis, and children’s academic achievements in these families are compared with those of children from intact families, with advantages consistently observed for the latter [7,8,17].

Another research strategy involves examining specificities of children from different types of non-intact families, comparing them with children from intact families. At the same time, most studies compare children from two-parent biological families with one of the typical variants of a single-parent family – either with single mothers or with children who experienced their parents’ divorce and rarely compare children from different types of non-intact families on academic achievement and related motivational variables. These latter studies point to specific differences between various non-intact families, although the findings remain inconsistent. For example, some studies show that adolescents living in stepparent families may be more vulnerable in terms of academic achievement and well-being than adolescents from other non-intact families [6]. A study based on a representative sample of Korean adolescents found that compared with adolescents from intact families, academic achievement was consistently lower among adolescents raised by a single-mother, a single-father, and those living in stepparent families [6]. Also compared with children raised in single-mother families created by the death of the father, children raised in divorced single-mother families have significantly lower levels of education and occupational status in adulthood [18]. On the other hand, a study of life satisfaction among adults who grew up in intact families, in single-mother families throughout childhood until age 15, or who lived part of their childhood with both parents followed by parental separation or divorce showed that the lowest levels of life satisfaction were observed among adults raised in single-mother families [14].

These findings support the importance of examining potentially different developmental conditions associated with these family structures and their distinct social situations of development, which may be associated with different outcomes [19].

### Mediators of the association between family structure and adolescents’ academic achievement

Despite evidence of lower academic achievements among children and adolescents from single-parent and stepparent families, it remains unclear what underlies these differences and which internal and external factors may account for them. In the context of examining psychological factors that mediates these associations, particular attention has been paid to the role of motivation and persistence, on the one hand, and satisfaction with family relationships, on the other.

Intrinsic and autonomous academic motivation, as well as persistence, are among the most extensively studied and reliable psychological predictors of academic success [20,21]. However, in the context of family structure, only a limited number of studies have examined motivation and academic engagement, which does not yet allow for a clear understanding of the psychological mechanisms underlying the association between family structure and academic achievement. For example, one longitudinal study showed that, compared with their peers from intact families, adolescents raised by single mothers exhibited declines over a five-year period in achievement motivation, competitiveness, desire for mastery, persistence, and willingness to endure negative consequences, with these effects being particularly pronounced among boys growing up without fathers [22]. A recent Russian study demonstrated that adolescents from non-intact families—including single-parent families, stepparent families, and extended families—showed lower levels of school engagement and higher levels of disengagement compared with adolescents from intact families, regardless of the number of children in the family [23].

On the other hand, other important factors related to academic motivation and academic achievement may include satisfaction with family relationships, family support and parenting style [24]. For example, it has been shown that adolescents from single-parent and stepparent family structures (combined sample), compared with adolescents from intact families, perceive lower levels of parental support, which, in turn, is associated with lower life satisfaction among adolescents [17].

In addition, single-mothers are more likely than cohabitating mothers to engage in psychologically controlling parenting behaviors, which predict higher levels of externalizing disorders and depressive symptoms among their adolescent children [25]. At the same time, several studies indicate that warm family relationships and family support may buffer developmental risks and lower levels of stress among children and adolescents growing up in non-intact families [7].

An important factor shaping the understanding of the role of family structure in the psychological development of children and adolescents is culture, which defines maternal and paternal roles in childrearing, societal tolerance toward single mothers, and state social policies aimed at supporting them [1,26]. Family structure distribution in different European countries and Canada shows wide variation across countries, ranging from 48% of 11–15 y.o. adolescents living with both parents in Azerbaijan to 92% in Albania, 73% in the total sample and 69% in Russia [17].

A recent ethnographic study demonstrated significant cross-cultural differences in childcare provided by maternal and paternal grandparents [27]. Specifically, the results showed that Russian children receive more care from maternal grandparents than from paternal grandparents, whereas in U.S. families the opposite pattern is observed, with paternal grandparents being more involved in childcare than maternal grandparents, and in Brazilian families, a relatively low level of involvement in childcare was observed among grandparents from both sides of the family. These findings raise the question of the specificity of the effects of growing up in single-mother family structures with motivational outcomes among Russian children and, in particular, allow for the assumption of weaker negative effects associated with living with a single mother.

### The present study

The present study primarily examines differences among adolescents from intact, single-mother, and stepfather families in academic motivation, homework motivation, academic persistence, academic achievement, homework cheating, and family satisfaction. These three family structures were selected because they are the most common in Russia and worldwide. Satisfaction with family is conceptualized as adolescents’ subjective perception and evaluation of their family relationships; high satisfaction indicates that adolescents are satisfied with their relationships, their communication with family members and feel happy within the family environment. More specifically, the main aim of the present study is to identify psychological factors underlying the association between family structure and academic achievement among Russian adolescents. The theoretical framework of the study is grounded in Vygotsky’s concept of the social situation of development [19] and Bronfenbrenner’s ecological approach [28], as applied to family-related factors in adolescent successful development.

We hypothesize, first, that family structure is associated with academic motivation and academic achievement, such that adolescents from intact families demonstrate more favorable levels of autonomous motivation, persistence, attitudes toward school, and academic achievement, as well as lower levels of homework cheating, than adolescents from single-mother and stepfather families. Second, we hypothesize that the association between family structure and adolescents’ autonomous academic motivation and academic achievement is partially mediated by satisfaction with family relationships.

## Materials and methods

### Participants and procedure

Participants were adolescents aged 10–16 years (M_age_ = 12.99; SD = 1.54; 51% girls, 49% boys), enrolled in grades 5–9. There were 431 students in Grade 5 (18%), 535 in Grade 6 (22%), 522 in Grade 7 (22%), 444 in Grade 8 (18%), and 470 in Grade 9 (20%). They were recruited from 12 public secondary schools in Moscow between December 2024 and March 2025. Schools returned a registration form indicating their intention to participate in the study. Schools informed parents about the survey by email and sent a text-message notification drawing their attention to the email. Parents could decline their child’s participation. Those who agreed provided written informed consent before data collection.

Data collection was conducted in school classrooms by two qualified psychologists. Participants completed paper-and-pencil questionnaires during a class period allocated by each school for the study. The survey was completed anonymously. All students who were present at school on the day of data collection and whose legal guardians had provided written informed consent were invited to participate. The adolescents were provided with age-appropriate information about the study and were informed that participation was voluntary and that they could decline to participate or withdraw from the study at any time without any negative consequences. The students also provided written assent on the first page of the questionnaire by confirming that they agreed to participate in the study.

A total of 3,047 questionnaires were distributed. After removing invalid responses (with >20% missing items, straight-lining) and responses from participants who did not meet the study criteria the sample size was 2,402. This final dataset contained no missing data on the key variables of interest; hence, missing data imputation was not applicable. All analyses were conducted on the complete case sample. The sample was divided into three groups according to the three characteristic types of family structure: an intact family (N = 1,757; 73%), a stepfather family (N = 180; 8%) and a single-mother family group (N = 465; 19%).

This study was approved by the Ethics Committee of the Faculty of Psychology at Lomonosov Moscow State University (the approval no: 2024/19) and conducted in accordance with relevant guidelines and the Declaration of Helsinki.

### Measures

#### Family structure.

Family structure was assessed using a self-report item: “Please indicate who you currently live with.” Response options included mother, father, stepmother, stepfather, and other.

#### Satisfaction with family.

We assessed adolescents’ satisfaction with family via the subscale from the Multidimensional Students’ Life Satisfaction Scale (MSLSS) [29,30]. However, this scale was slightly modified. We selected only those statements (4 items) that describe positive, supportive relations in the family: “I like spending time with my parents”, “My family gets along well together”, “I enjoy being at home with my family”, “My parents and 1 do fun things together”. Participants rated their answers on a response scale ranging from 1 to 5. The single-factor model of this scale demonstrated acceptable fit: χ^2^ = 35.79; df = 2; p < 0.001; CFI = 0.984; TLI = 0.951; SRMR = 0.020; RMSEA = 0.084 (90% CI = [0.061, 0.109]); PCLOSE = 0.008; N = 2402.

#### Academic motivation.

To assess academic motivation, we used a Universal Academic Motivation Scale (UAMS) that we developed based on previous similar scales [31,32] based on self-determination theory [33]. The questionnaire allows assessing two types of autonomous academic motivation: intrinsic (6 items including motivation to know and achievement motivation) and identified motivation (3 items), two types of controlled motivation: introjected motivation (3 items) and external motivation (3 items), as well as amotivation (3 items). The scales of intrinsic, identified motivation and amotivation had good internal consistency (Table 1), while for the scales of introjected and external motivation, the internal consistency coefficients were moderate but acceptable for research purposes (0.61–0.63). This study focuses on autonomous motivation, which demonstrated high reliability. The five-factor CFA model for this questionnaire indicated excellent fit: χ^2^ = 573.65; df = 119; p < 0.001; CFI = 0.963; TLI = 0.952; RMSEA = 0.040 (90% CI = [0.037, 0.043]); PCLOSE = 1. The model included covariances between the items for motivation to know and achievement motivation within the intrinsic motivation factor.

**Table 1:** Descriptive statistics, reliabilities and Pearson's r correlations among the study variables.

Variables	M	SD	α	1	2	3	4	5	6	7	8	9	10	11	12	13	14	15	16	17	18
**1. Autonomous motivation**	3.44	0.83	0.87	—																	
**2. Intrinsic motivation**	3.17	0.96	0.87	0.95*	—																
**3. Identified motivation**	3.98	0.91	0.71	0.75*	0.51*	—															
**4. Introjected motivation**	3.11	1.06	0.61	0.26*	0.19*	0.31*	—														
**5. External motivation**	2.91	1.09	0.63	−0.12*	−0.15*	−0.02	0.32*	—													
**6. Amotivation**	1.94	0.97	0.73	−0.53*	−0.43*	−0.55*	−0.08*	0.16*	—												
**7. Academic persistence**	2.90	0.57	0.75	0.65*	0.62*	0.48*	0.14*	−0.18*	−0.48*	—											
**8. Autonomous homework motivation**	3.25	0.98	0.86	0.75*	0.67*	0.65*	0.25*	−0.11*	−0.51*	0.67*	—										
**9. Introjected homework motivation**	2.93	1.17	0.76	0.34*	0.26*	0.37*	0.60*	0.22*	−0.20*	0.27*	0.41*	—									
**10. External homework motivation**	2.59	1.07	0.73	−0.10*	−0.13*	−0.01	0.25*	0.61*	0.15*	−0.16*	−0.05	0.31*	—								
**11. Homework persistence**	2.95	0.65	0.68	0.51*	0.47*	0.40*	0.14*	−0.14*	−0.43*	0.73*	0.57*	0.28*	−0.09*	—							
**12. Homework cheating**	2.18	0.85	0.84	−0.45*	−0.42*	−0.36*	−0.08*	0.13*	0.40*	−0.53*	−0.48*	−0.24*	0.08*	−0.58*	—						
**13. GPA**	4.26	0.48	0.89	0.36*	0.34*	0.26*	0.14*	−0.06*	−0.25*	0.37*	0.29*	0.21*	−0.04	0.34*	−0.34*	—					
**14. Satisfaction with family**	4.02	0.90	0.83	0.31*	0.28*	0.26*	0.01	−0.29*	−0.27*	0.40*	0.35*	0.10*	−0.30*	0.36*	−0.28*	0.13*	—				
**15. Satisfaction with school**	2.86	0.89	0.83	0.60*	0.58*	0.44*	0.11*	−0.16*	−0.45*	0.56*	0.67*	0.24*	−0.13*	0.48*	−0.41*	0.24*	0.32*	—			
**16. Satisfaction with teachers**	3.30	0.96	0.89	0.52*	0.49*	0.41*	0.13*	−0.12*	−0.33*	0.48*	0.55*	0.25*	−0.10*	0.43*	−0.35*	0.24*	0.37*	0.64*	—		
**17. Attitude toward school**	4.58	1.49	—	0.61*	0.57*	0.49*	0.17*	−0.14*	−0.49*	0.55*	0.65*	0.28*	−0.11*	0.45*	−0.39*	0.28*	0.29*	0.67*	0.53*	—	
**18. Attitude toward class**	4.98	1.61	—	0.26*	0.25*	0.18*	0.06*	−0.07*	−0.19*	0.28*	0.29*	0.11*	−0.05	0.24*	−0.18*	0.12*	0.24*	0.35*	0.31*	0.37*	—
**19. Grade**	6.99	1.38	—	−0.03	0.01	−0.11*	0.00	−0.02	0.09*	−0.13*	−0.13*	−0.14*	−0.16*	−0.25*	0.28*	−0.17*	−0.10*	−0.13*	−0.06	−0.09*	−0.02

M — means, SD — standard deviations, α — Cronbach’s alpha, * — correlations are signiﬁcant at p < 0.01.

#### Homework motivation.

Homework Motivation Questionnaire was used to measure another type of academic motivation related to homework [34] also based on self-determination theory. It consists of 13 statements that complete the stem phrase (“I do my homework because…”) and form three scales: autonomous motivation (6 items, e.g., “I like to learn and be able to do more and more”), introjected motivation (3 items, e.g., “I’m ashamed to get bad grades”), and external motivation (4 items, e.g., “I’m not allowed to do anything else until it is done”). The autonomous motivation scale includes three pairs of statements measuring motivation to know, achievement motivation (intrinsic motivation), and identified motivation. All scales had good internal consistency (Table 1). A three-factor model (with covariances between the pairs of items representing the subtypes of autonomous motivation) indicated acceptable fit: χ^2^ = 782.86; *df* = 62; p < 0.001; CFI = 0.921; TLI = 0.900; RMSEA = 0.070 (90% CI = [0.065, 0.074]); PCLOSE = 0. We used a five-point Likert-type response scale in these motivation measures.

#### Academic persistence.

We employed Academic Persistence Scale, which consists of seven items (e.g., “I like to make an effort in my studies”) [35] with a 4-point Likert-type response scale. Additionally, we used the Homework Persistence Scale, which included four items, two of which were reversed (e.g., “I can’t work long and hard on my homework”) [34,36].

#### Homework cheating.

Homework cheating was assessed using three items (“I sometimes cheat on my homework, as many students do”, “Sometimes I just cheat on my homework because I don’t have time to do it at home”, “I just cheat on my homework using the Internet and neural networks”). Students rated their agreement with each item using a 4-point Likert scale [34,36].

#### Academic achievement.

Students’ academic achievement was evaluated via self-reported grades on 12 school subjects (all subjects except physical education, music, handicraft and drawing) for the last term. Grades from the last term were used because recent grades were expected to be recalled more accurately than grades from earlier terms, thereby minimizing potential recall-related distortion. GPA was calculated as the average of these 12 grades for each student.

#### Satisfaction with school and teachers.

Satisfaction with school and teachers was assessed via corresponding subscales from the Multidimensional Students’ Life Satisfaction Scale (MSLSS), Russian version [29,30]. As an additional indicator of satisfaction with school, a nonverbal measure of general attitude towards school and class was employed [37]. It depicts seven faces, ranging from most happy to least happy. The participant was asked to decide which face best reflects his/her attitude towards school and class.

### Data analysis

We ﬁrst generated descriptive statistics and correlations for all studied variables and assessed gender differences using Welch t-test (in R environment). Next, we estimated the effects of three types of family structure on studied variables using Welch one-way ANOVA and Games-Howell post-hoc test. To assess the effect size, we used ω^2^ (using WAnova package for R environment), which provides an estimation of the explained variance, but is less biased than η^2^. Interpretation of ω^2^ value was based on the following cut-offs: 0.01 — small, 0.06 — medium, 0.14 — large [38].

To test the hypothesis of indirect effects, we conducted structural equation modeling in Mplus 8 using maximum likelihood estimator with robust standard deviations and Yuan-Bentler statistic χ^2^ (MLR). All path coefficients were standardized. The coefficients for binary variables were standardized by dependent variable (STDY option in Mplus) so their values indicate the difference between the two levels of independent variable in standard deviation of dependent variable. Bootstrap analysis (5,000 resamples) was used to assess the statistical significance of indirect effects. The data and code are available in the online supplement (https://osf.io/bkgys/).

## Results

Table 1 reports the means, standard deviations, and correlations between all measured variables. In this table, we can see the expected pattern of positive correlations of GPA with indicators of autonomous motivation, persistence, and satisfaction with family as well as a similar pattern of negative correlations for homework cheating.

The age group (school grade) was negatively correlated with the identified motivation, persistence, every scale of homework motivation, homework persistence, GPA, satisfaction with family, school, teachers and attitude toward school. Positive correlations were found for amotivation and cheating.

We also revealed gender differences on study variables: girls had higher identified and introjected academic motivation, homework introjected motivation, homework cheating and GPA (all *p* < 0.01, see S1 Table for details). Boys scored higher on intrinsic, autonomous, and external academic motivation, academic persistence, homework external motivation and persistence, as well as on satisfaction with family, teachers, and positive attitude toward class (all p < 0.01), but the effect sizes in each case were weak (Cohen’s d < 0.25). Thus, age group and gender were important variables related to many others, and their effects needed to be controlled during further structural equation modeling.

Table 2 reports the results of one-way Welch ANOVA. Compared with the other families, adolescents from intact families had significantly higher autonomous (including intrinsic and identified) academic motivation, homework autonomous motivation, academic persistence and homework persistence, GPA as well as lower amotivation and homework cheating. Also, adolescents from intact families scored higher on satisfaction with family, school, positive attitude to school and class. Post-hoc comparisons indicated that adolescents from single-mother and stepfather families did not differ significantly on any of the variables (see superscripts in Table 2), having significantly less favorable mean values in comparison to intact families. All these effects were weak by their magnitude (ω^2^ < 0.014).

**Table 2:** Differences in motivation, persistence, academic achievement, and satisfaction with family among adolescents from different family structures.

Variables	Intact		Single mother		Stepfather		Welch F(2;df)	df	p	Effect size ω^2^
	M	SD	M	SD	M	SD				
**Autonomous motivation**	3.48^a^	0.83	3.36^b^	0.83	3.22^b^	0.83	11.32	428.5	<.001	0.009
**Intrinsic motivation**	3.22^a^	0.95	3.09^b^	0.95	2.91^b^	0.97	10.28	426.8	<.001	0.008
**Identified motivation**	4.02^a^	0.90	3.90^b^	0.91	3.82^b^	0.94	6.33	424.5	<.01	0.004
**Introjected motivation**	3.13	1.06	3.08	1.08	3.04	1.02	1.02	431.5	0.363	0.000
**External motivation**	2.93	1.09	2.85	1.10	2.94	1.13	1.10	424.4	0.334	0.000
**Amotivation**	1.92^a^	0.95	1.95^ab^	1.00	2.13^b^	1.06	3.50	417.2	<.05	0.002
**Academic persistence**	2.94^a^	0.57	2.80^b^	0.59	2.81^b^	0.54	12.15	430.5	<.001	0.009
**Autonomous homework motivation**	3.32^a^	0.96	3.11^b^	1.03	2.99^b^	0.99	14.83	422.8	<.001	0.011
**Introjected homework motivation**	2.96	1.16	2.85	1.20	2.77	1.24	3.13	421.4	<0.05	0.002
**External homework motivation**	2.62	1.06	2.52	1.10	2.53	1.11	1.82	422.4	0.164	0.001
**Homework persistence**	2.98^a^	0.64	2.86^b^	0.67	2.84^b^	0.67	8.82	422.5	<.001	0.006
**Homework cheating**	2.13^a^	0.84	2.31^b^	0.88	2.38^b^	0.86	13.81	423.8	<.001	0.011
**GPA**	4.29^a^	0.47	4.17^b^	0.48	4.20^b^	0.45	13.39	431.8	<.001	0.010
**Satisfaction with family**	4.09^a^	0.85	3.83^b^	1.01	3.84^b^	0.99	16.96	409	<.001	0.013
**Satisfaction with school**	2.89^a^	0.89	2.81^ab^	0.87	2.66^b^	0.83	7.11	437.5	<.01	0.005
**Satisfaction with teachers**	3.33	0.97	3.27	0.97	3.18	0.90	2.62	435.4	0.074	0.001
**Attitude toward school**	4.62^a^	1.48	4.50^ab^	1.53	4.33^b^	1.49	3.88	426.2	<.05	0.002
**Attitude toward class**	5.06^a^	1.57	4.84^b^	1.68	4.58^b^	1.79	8.11	414.6	<.001	0.006

M — means, SD — standard deviations.

Letters in superscripts illustrate the results of Games-Howell post-hoc test. The same letter in two groups means that they are homogeneous, different letters indicate on statistically significant differences (*p* ≤ 0.05). Two letters in superscripts (“ab”) means that this group do not significantly differ from either the group “a” or the group “b”, while groups “a” and “b” differ significantly from each other. The absence of a letter indicates that there are no significant differences.

Next, we tested a structural equation model to analyze the whole pattern of relations between family structure, satisfaction with family, indicators of motivation, persistence, cheating and academic achievement. This model included two indicators of motivation (academic autonomous motivation and autonomous homework motivation), two indicators of persistence (academic persistence and homework persistence) and two indicators of academic achievement (GPA and homework cheating as a reverse indicator). Motivation and persistence were expected to be directly related to satisfaction with family and mediated its effect on academic achievement. Direct paths from family satisfaction to GPA and homework cheating were fixed at 0. In addition, we included type of family structure, gender and grade with all their possible effects on all the other variables. We coded the type of family structure as binary variable (1 — intact family, 0 — single mother or stepfather families) because the results of ANOVA indicated on the homogeneity of the samples from single-mother and stepfather families by all variables included in the model.

This model indicated an excellent ﬁt (χ^2^ = 7.36; df = 2; p = 0.025; CFI = 0.999; TLI = 0.984; RMSEA = 0.033 (90% CI = [0.010, 0.061]); PCLOSE = 0.821), however we found a negative suppression effect [39] in the results, which distorted the true relationships between variables. The path coefficient of homework autonomous motivation on GPA (–0.12; *p* < 0.001) had the opposite sign to the corresponding zero-order correlation (0.29; *p* < 0.001). To solve this problem, we fixed the path between these variables at zero in accordance with a psychologically plausible assumption of a fully mediated (via homework persistence) relationship between autonomous homework motivation and GPA.

**Fig 1:**
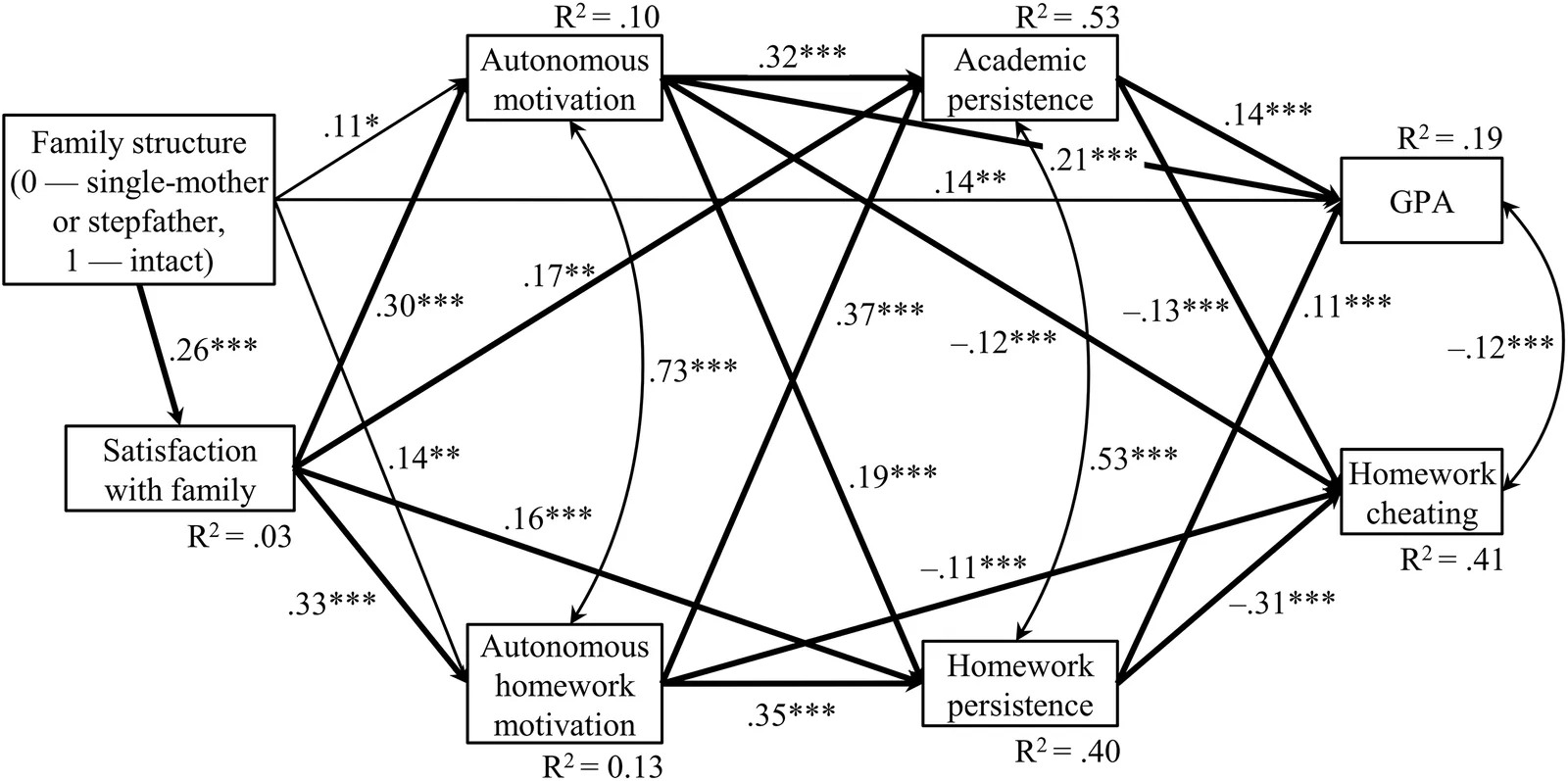
Structural equation model of relations among the family type, satisfaction with family, motivation and persistence to predict GPA and homework cheating. Controlled variables “gender” and “grade” along with their effects and insignificant paths are omitted for parsimony, all the coefficients are standardized; * *p* < 0.05, ** *p* < 0.01, *** *p* < 0.001. All tested and significant mediation paths via satisfaction with family are indicated by weighted lines.

This last model (Fig 1) also indicated an excellent ﬁt: χ^2^ = 19.92; df = 3; p < 0.001; CFI = 0.998; TLI = 0.967; RMSEA = 0.048 (90% CI = [0.030, 0.070]); PCLOSE = 0.505. As the effects of grade and gender were not the focus of this study, they (along with the corresponding paths) were omitted from Fig 1 for parsimony (see S2 Table for details).

The model presented in Fig 1 demonstrates that family structure had statistically significant direct effects not only on family satisfaction, but also on autonomous motivation, as well as academic performance. At the same time, we revealed statistically significant indirect effects of family structure (mediated by family satisfaction) on motivation and achievement indicators: in particular the effects of family structure on academic autonomous motivation (0.08; p ≤ 0.001; 95% CI [0.05, 0.11]) and autonomous homework motivation (0.09; p ≤ 0.001; 95% CI [0.05, 0.12]). We also found the overall statistically significant indirect effect of family structure (via family satisfaction and then via the other variables) on GPA (0.04; p ≤ 0.001; 95% CI [0.03, 0.06]) and homework cheating (–0.06; p ≤ 0.001; 95% CI [–0.08, –0.04]). All specific indirect paths were also statistically significant (see S3 Table for details). However, given their negligible magnitude (all < 0.01), they are not discussed here. So, family satisfaction proved to be a mediator of the relationship between family structure and indicators of autonomous motivation, persistence and academic achievement, however this mediation effect is only partial.

## Discussion

Previous research indicates that family structure is associated with students’ academic achievement: children from intact families demonstrate higher academic achievement than children from single-mother and stepfather families. However, there is still a limited number of studies that examine the psychological mediators of the association between family structure and academic achievement. A key novelty lies in examining, within a single model, family-related and motivational psychological factors that account for differences in academic achievement among adolescents from different family structures.

Our findings support the first hypothesis and indicate that family structure is associated with satisfaction with family and autonomous motivation, which, in turn, is associated with persistence and academic achievement among adolescents. Specifically, adolescents from intact families demonstrated higher levels of satisfaction with family, autonomous academic motivation, autonomous motivation for doing homework, academic persistence, homework persistence, academic achievement, and positive attitude toward class, as well as lower levels of homework cheating, compared with adolescents from single-mother and stepfather families. No significant differences were found between adolescents from single-mother and stepfather families.

The similar levels of satisfaction with family, autonomous motivation, persistence, and academic achievement observed among adolescents from single-mother families, which did not differ from those demonstrated by adolescents from stepfather families with two adults, may be related to a characteristic feature of Russian families—greater involvement and care provided by maternal relatives compared with paternal relatives [27]. In this context, maternal relatives may support the single mother, not leaving her alone in caring for the child.

At the same time, analyses by family structure show that adolescents from single-mother and stepfather families report significantly lower satisfaction with family than adolescents from intact families, whereas no differences are observed in satisfaction with teachers. The absence of differences in satisfaction with teachers and external motivation, as well as the relatively weak differences in attitudes toward school, may be related to a favorable school climate, which may serve as a protective factor buffering the associations between non-intact family structure and adolescents’ academic achievement. This assumption is supported by previous research demonstrating that a positive school climate can substantially attenuate the negative effects of family structure on the academic achievement of adolescents from non-intact families [5] and on their externalizing problems [40].

Among studies reporting advantages in academic achievement for adolescents from intact families compared with adolescents from single-parent and stepparent families, a simultaneous analysis of all three family structures has been presented only in the study by Park and Lee [6]. In contrast, the findings of the present study did not reveal significant differences between adolescents from single-parent and stepparent families, whereas the results of the Korean study indicate lower academic achievement among adolescents from stepparent families compared with those from single-parent families. At the same time, in both studies adolescents from intact families demonstrated higher levels of academic achievement. When interpreting these discrepancies, it is essential to consider the sociocultural context. In South Korea, single-parent and stepparent families remain relatively rare, whereas in the Russian context these family structures are common and widespread [2] and both types of non-intact families can be perceived by children as equally “normal”.

Our findings also support our second hypothesis that the association between family structure and adolescents’ autonomous academic motivation and academic achievement is mediated by satisfaction with family relationships. The indirect effects confirm that satisfaction with family relationships partially mediates the associations between family structure and autonomous motivation, which, in turn, is associated with adolescents’ persistence, homework cheating, and academic achievement. From the perspective of self-determination theory, satisfaction with family relationships may reflect the fulfillment of adolescents’ basic psychological needs for autonomy, competence, and relatedness, which are considered the primary sources of intrinsic motivation in academic activities [41].

However, this mediation is partial: alongside the expected indirect effects, direct effects were also observed. The direct effect of family structure on adolescents’ autonomous academic motivation, autonomous motivation for doing homework, and academic achievement may be explained by two factors. First, one possible interpretation is that mothers in single-mother families may themselves represent less successful educational role models, demonstrating lower interest in the process of learning and in the values of intellectual development. This interpretation is consistent with previous research showing that single-mother families are characterized by relatively lower parental educational attainment [18], longer and more demanding working hours, and lower income [3,18,42]. Second, these characteristics of single-mother families may be associated with a less diverse developmental environment and more limited access to supplementary educational opportunities, which may constrain adolescents’ intellectual development. In stepfather families, by contrast, the direct effect of family structure on motivation and academic achievement may partly reflect reduced maternal time available for communication and academic support, as mothers may be engaged in building new relationships.

Finally, common factors across both non-intact family structures that may account for the direct negative effects of family structure on motivation and academic achievement include reduced attention to adolescents’ needs, more superficial discussions of school-related issues and homework [13], as well as negative experiences associated with having a family structure that differs from that of most peers and with overall family stress [6].

The findings of the present study extend our understanding of adolescents’ academic achievement in the context of family functioning and contribute to the existing literature in this field. The study was not intended to be exhaustive; rather, its aim was to highlight the importance of SEM analysis to understand the system of relationships between family structure, satisfaction with family, and motivational variables in terms of their predictive role in adolescents’ academic achievement. It is possible that the observed findings regarding the role of family structure in adolescents’ autonomous motivation and its association with academic achievement are culturally specific and therefore require replication in samples from other cultural contexts. In this respect, the present findings are not fully consistent with results from a Canadian longitudinal study, which showed that family structure (configuration) in early childhood was not associated with classroom engagement in primary school or adolescence [43].

Note that academic achievement in the present study was assessed using self-reported grades, which should be considered when interpreting the findings. Nevertheless, previous research has shown that self-reported grades are highly positively correlated with actual grades across school subjects, supporting their reliability as an indicator of academic achievement [44,45].

Thus, the findings of the present study have several practical implications. In particular, greater emphasis may be placed on supporting parents in single-mother and stepfather families in creating a favorable psychological climate that may contribute to adolescents’ greater satisfaction with family relationships. Psychological work with parents, provided by school psychologists or specialists at family support centers, may include parent education programs that promote positive, need-supportive parenting practices and are tailored to the specific characteristics and challenges associated with different family structures. Such programs may help foster family relationships that support adolescents’ basic psychological needs.

### Limitations and strengths

The present study is not without limitations. First, the cross-sectional design does not allow for causal inferences. Analyses based on cross-sectional questionnaire data can only suggest associations among variables, but not causation [46]. Longitudinal research can help analyze the causal effect of family structure and family satisfaction on motivation and academic achievement. Nevertheless, our empirical findings align with theoretical predictions concerning the probable effects of family structure and the quality of family relationships on adolescents’ autonomous motivation and achievement [19,33]. Second, the study lacked information about when the family became single-mother or stepfather, which makes it impossible to determine whether a single-mother family existed from the outset and how much time had passed since parental separation or divorce. However, at the study design stage, we made a deliberate decision not to ask adolescents detailed questions about family transitions to avoid potential distress, in line with ethical considerations. Third, the satisfaction with family scale used in the Huebner scale (MSLSS) allows for the assessment of adolescents’ satisfaction with family relationships but does not provide information about specific parenting practices or parenting styles. Fourth, the study was originally designed to examine the motivational characteristics of adolescents from intact families in comparison with those from single-mother and stepfather families. This focus limits the generalizability of the findings to other family structures. Finally, the study did not control for family socioeconomic status (SES). Accounting for SES would have allowed a more precise assessment of the satisfaction with family relationships as a mediator of the associations of family structure with motivational variables and academic achievement.

This study has several chief strengths that provide a well-rounded picture of family factors, motivational factors, including interest in learning and persistence, and academic achievement as described by Symonds et al. [11]. First, this study is the first to examine differences in academic motivation, motivation for homework, and persistence across different family structures in a sample of Russian adolescents using validated and culturally adapted instruments. The use of psychometrically sound measures enhances the reliability of the observed associations. Second, the use of a large, representative sample of Russian adolescents, combined with structural equation modeling, allowed us to obtain robust estimates of both direct and indirect effects of family structure and satisfaction with family on adolescents’ academic motivation and academic achievement. Third, an additional strength of the study lies in moving beyond comparisons between intact families and combined non-intact family groups. The study further examined differences between specific non-intact families, namely single-mother and stepfather families, with respect to academic motivation, persistence, academic achievement, and homework cheating. Future research should focus on a more detailed examination of the reasons underlying lower satisfaction with family in single-parent and stepparent families, as well as on the role of supportive parenting styles, particularly autonomy support and reduced psychological and behavioral control [47], in relation to more favorable outcomes among adolescents from non-intact families. Further studies should also examine the extent to which the observed patterns apply to adolescents from other family types (e.g., single-father families, grandparent-headed families, and foster families).

## Conclusion

The findings of the present study indicate that lower satisfaction with family among adolescents from single-mother and stepfather families mediates the association between family structure and reduced autonomous academic motivation, persistence, homework cheating, and academic achievement. At the same time, adolescents’ satisfaction with family also has an independent association with autonomous motivation, interest in learning, and academic achievement.

The findings further demonstrate similarities between adolescents from single-mother and stepfather families in their levels of satisfaction with family, autonomous academic motivation, and academic achievement, suggesting that for adolescents’ intrinsic motivation, persistence, and academic achievement, not the number of adults in the family but the quality of family relationships is important.

Overall, the findings substantially extend previous evidence on the system of family-related factors, including the role of family structure in the successful development of children and adolescents, and highlight the importance of developing psychological support programs for families. Such programs may focus on enhancing effective parental communication and supporting adolescents’ basic psychological needs for relatedness, competence, and autonomy, as well as on providing teachers with recommendations for supporting the motivation of adolescents from at-risk groups.

The practical implications of the findings underscore the importance of attention from both schools and society not only to adolescents growing up with single mothers, but also to those from stepparent families, who may represent potential risk groups for school demotivation and academic underachievement.

## Supporting information

S1 Table Gender differences on study variables. (DOCX)

S2 Table SEM results. Standardized Model Results (STDY Standardization, Fit indexes: χ2 = 19.92; df = 3; p < 0.001; CFI = 0.998; TLI = 0.967; RMSEA = 0.048 (90% CI = [0.030, 0.070]); PCLOSE = 0.505; N = 2402).
(DOCX)

S3 Table SEM results. Indirect effects estimates (Standardized Model Results, STDY Standardization). (DOCX)
