# Supplementary material for: Family structure and family satisfaction as factors of adolescents’ autonomous motivation, cheating, and academic achievement
Source: PLoS One. 2026 Sep 16;21(9):e0357562. doi: 10.1371/journal.pone.0357562 (PMC13580952; doi:10.1371/journal.pone.0357562)
Supplement: Supplementary file 1 [file pone.0357562.s001.docx]

**S1 Table. Gender differences on study variables.**

| Variables | Mean (F) | Mean (M) | t | df | p | Cohen's d | CI lower | CI upper |
| --- | --- | --- | --- | --- | --- | --- | --- | --- |
| **Autonomous motivation** | 3.40 | 3.49 | –2.65 | 2372.93 | 0.008 | –0.11 | –0.19 | –0.03 |
| **Intrinsic motivation** | 3.06 | 3.29 | –5.96 | 2393.02 | 0.000 | –0.24 | –0.32 | –0.16 |
| **Identified motivation** | 4.08 | 3.88 | 5.26 | 2308.80 | 0.000 | 0.22 | 0.14 | 0.30 |
| **Introjected motivation** | 3.24 | 2.99 | 5.88 | 2379.51 | 0.000 | 0.24 | 0.16 | 0.32 |
| **External motivation** | 2.85 | 2.98 | –2.93 | 2397.79 | 0.003 | –0.12 | –0.20 | –0.04 |
| **Amotivation** | 1.91 | 1.97 | –1.61 | 2383.92 | 0.108 | –0.07 | –0.15 | 0.01 |
| **Academic perseverance** | 2.86 | 2.94 | –3.09 | 2397.14 | 0.002 | –0.13 | –0.21 | –0.05 |
| **Homework autonomous motivation** | 3.25 | 3.25 | 0.02 | 2365.97 | 0.984 | 0.00 | –0.08 | 0.08 |
| **Homework introjected motivation** | 3.01 | 2.84 | 3.46 | 2392.84 | 0.001 | 0.14 | 0.06 | 0.22 |
| **Homework external motivation** | 2.52 | 2.67 | –3.58 | 2387.34 | 0.000 | –0.15 | –0.23 | –0.07 |
| **Homework perseverance** | 2.90 | 3.00 | –3.75 | 2399.20 | 0.000 | –0.15 | –0.23 | –0.07 |
| **Homework cheating** | 2.25 | 2.11 | 3.90 | 2397.70 | 0.000 | 0.16 | 0.08 | 0.24 |
| **GPA** | 4.29 | 4.23 | 3.13 | 2378.86 | 0.002 | 0.13 | 0.05 | 0.21 |
| **Satisfaction with family** | 3.95 | 4.10 | –4.14 | 2343.08 | 0.000 | –0.17 | –0.25 | –0.09 |
| **Satisfaction with school** | 2.85 | 2.86 | –0.41 | 2375.40 | 0.684 | –0.02 | –0.10 | 0.06 |
| **Satisfaction with teachers** | 3.21 | 3.40 | –4.86 | 2394.96 | 0.000 | –0.20 | –0.28 | –0.12 |
| **Attitude to school** | 4.63 | 4.53 | 1.58 | 2364.61 | 0.115 | 0.06 | –0.02 | 0.14 |
| **Attitude to class** | 4.85 | 5.12 | –4.08 | 2399.54 | 0.000 | –0.17 | –0.25 | –0.09 |
