# Supplementary material for: Family structure and family satisfaction as factors of adolescents’ autonomous motivation, cheating, and academic achievement
Source: PLoS One. 2026 Sep 16;21(9):e0357562. doi: 10.1371/journal.pone.0357562 (PMC13580952; doi:10.1371/journal.pone.0357562)
Supplement: Supplementary file 2 [file pone.0357562.s002.docx]

**S2 Table. SEM results.**

| **Effects** | **DVs and mediators** | **Estimate** | **S.E.** | **Est./S.E.** | **P-Value** | **Lower 2.5%** | **Upper 2.5%** |
| --- | --- | --- | --- | --- | --- | --- | --- |
| **AUTMOT ON** | |  |  |  |  |  |  |
|  | **SATFAM** | 0,30 | 0,02 | 15,37 | 0,000 | 0,26 | 0,34 |
|  | **GENDER** | 0,05 | 0,04 | 1,29 | 0,196 | –0,02 | 0,13 |
|  | **GRADE** | 0,00 | 0,02 | 0,08 | 0,938 | –0,03 | 0,03 |
|  | **FAMTYPE** | 0,11 | 0,05 | 2,43 | 0,015 | 0,02 | 0,20 |
| **HWAUTMOT ON** | |  |  |  |  |  |  |
|  | **SATFAM** | 0,33 | 0,02 | 17,19 | 0,000 | 0,29 | 0,37 |
|  | **GENDER** | –0,07 | 0,04 | –1,85 | 0,065 | –0,15 | 0,00 |
|  | **GRADE** | –0,07 | 0,01 | –4,75 | 0,000 | –0,10 | –0,04 |
|  | **FAMTYPE** | 0,14 | 0,05 | 3,13 | 0,002 | 0,05 | 0,23 |
| **ACPERS ON** | |  |  |  |  |  |  |
|  | **AUTMOT** | 0,32 | 0,03 | 12,66 | 0,000 | 0,27 | 0,37 |
|  | **HWAUTMOT** | 0,37 | 0,02 | 15,37 | 0,000 | 0,32 | 0,42 |
|  | **SATFAM** | 0,17 | 0,02 | 10,16 | 0,000 | 0,13 | 0,20 |
|  | **GENDER** | 0,06 | 0,03 | 2,15 | 0,031 | 0,01 | 0,12 |
|  | **GRADE** | –0,04 | 0,01 | –3,51 | 0,000 | –0,06 | –0,02 |
|  | **FAMTYPE** | 0,01 | 0,03 | 0,39 | 0,698 | –0,05 | 0,08 |
| **HMPERS ON** | |  |  |  |  |  |  |
|  | **AUTMOT** | 0,19 | 0,03 | 7,41 | 0,000 | 0,14 | 0,25 |
|  | **HWAUTMOT** | 0,35 | 0,03 | 13,32 | 0,000 | 0,29 | 0,39 |
|  | **SATFAM** | 0,16 | 0,02 | 8,21 | 0,000 | 0,12 | 0,20 |
|  | **GENDER** | 0,10 | 0,03 | 3,08 | 0,002 | 0,04 | 0,16 |
|  | **GRADE** | –0,13 | 0,01 | –11,43 | 0,000 | –0,16 | –0,11 |
|  | **FAMTYPE** | –0,01 | 0,04 | –0,25 | 0,804 | –0,08 | 0,06 |
| **GPA ON** |  |  |  |  |  |  |  |
|  | **ACPERS** | 0,14 | 0,03 | 4,40 | 0,000 | 0,08 | 0,20 |
|  | **HMPERS** | 0,11 | 0,03 | 3,88 | 0,000 | 0,05 | 0,17 |
|  | **AUTMOT** | 0,21 | 0,03 | 7,88 | 0,000 | 0,15 | 0,26 |
|  | **GENDER** | –0,20 | 0,04 | –5,44 | 0,000 | –0,27 | –0,13 |
|  | **GRADE** | –0,08 | 0,01 | –5,98 | 0,000 | –0,11 | –0,05 |
|  | **FAMTYPE** | 0,14 | 0,04 | 3,41 | 0,001 | 0,06 | 0,22 |
| **CHEATING ON** | |  |  |  |  |  |  |
|  | **HMPERS** | –0,31 | 0,03 | –11,78 | 0,000 | –0,36 | –0,26 |
|  | **ACPERS** | –0,13 | 0,03 | –4,30 | 0,000 | –0,19 | –0,07 |
|  | **HWAUTMOT** | –0,11 | 0,03 | –3,67 | 0,000 | –0,16 | –0,05 |
|  | **AUTMOT** | –0,12 | 0,03 | –4,23 | 0,000 | –0,17 | –0,06 |
|  | **GENDER** | –0,07 | 0,03 | –2,20 | 0,028 | –0,13 | –0,01 |
|  | **GRADE** | 0,12 | 0,01 | 10,04 | 0,000 | 0,10 | 0,15 |
|  | **FAMTYPE** | –0,07 | 0,04 | –1,85 | 0,065 | –0,14 | 0,01 |
| **SATFAM ON** | |  |  |  |  |  |  |
|  | **GENDER** | 0,15 | 0,04 | 3,70 | 0,000 | 0,07 | 0,22 |
|  | **GRADE** | –0,06 | 0,02 | –4,26 | 0,000 | –0,09 | –0,03 |
|  | **FAMTYPE** | 0,26 | 0,05 | 5,40 | 0,000 | 0,16 | 0,36 |
| **AUTMOT WITH** | |  |  |  |  |  |  |
|  | **HWAUTMOT** | 0,73 | 0,01 | 66,02 | 0,000 | 0,70 | 0,75 |
| **ACPERS WITH** | |  |  |  |  |  |  |
|  | **HMPERS** | 0,53 | 0,02 | 31,40 | 0,000 | 0,50 | 0,57 |
| **CHEATING WITH** | |  |  |  |  |  |  |
|  | **GPA** | –0,12 | 0,02 | –5,64 | 0,000 | –0,16 | –0,08 |

Standardized Model Results (STDY Standardization, Fit indexes: χ2 = 19.92; df = 3; p < 0.001; CFI = 0.998; TLI = 0.967; RMSEA = 0.048 (90% CI = [0.030, 0.070]); PCLOSE = 0.505; N = 2402).
