# Supplementary material for: Family structure and family satisfaction as factors of adolescents’ autonomous motivation, cheating, and academic achievement
Source: PLoS One. 2026 Sep 16;21(9):e0357562. doi: 10.1371/journal.pone.0357562 (PMC13580952; doi:10.1371/journal.pone.0357562)
Supplement: Supplementary file 3 [file pone.0357562.s003.docx]

**S3 Table. SEM results. Indirect effects estimates (Standardized Model Results, STDY Standardization).**

| **Effects** | **DVs and mediators** | **Estimate** | **S.E.** | **Est./S.E.** | **P-Value** | **Lower 2.5%** | **Upper 2.5%** |
| --- | --- | --- | --- | --- | --- | --- | --- |
| **Effects from FAMTYPE to HWAUTMOT** | | | |  |  |  |  |
| **Total** |  | 0,228 | 0,047 | 4,849 | 0,000 | 0,135 | 0,317 |
| **Total indirect** | | 0,087 | 0,017 | 5,045 | 0,000 | 0,054 | 0,122 |
| **Specific indirect 1** | |  |  |  |  |  |  |
|  | **HWAUTMOT** | |  |  |  |  |  |
|  | **SATFAM** |  |  |  |  |  |  |
|  | **FAMTYPE** | 0,087 | 0,017 | 5,045 | 0,000 | 0,054 | 0,122 |
| **Direct** |  |  |  |  |  |  |  |
|  | **HWAUTMOT** | |  |  |  |  |  |
|  | **FAMTYPE** | 0,141 | 0,045 | 3,102 | 0,002 | 0,051 | 0,230 |
| **Effects from FAMTYPE to AUTMOT** | | | |  |  |  |  |
| **Total** |  | 0,187 | 0,047 | 4,028 | 0,000 | 0,097 | 0,278 |
| **Total indirect** | | 0,078 | 0,016 | 4,974 | 0,000 | 0,049 | 0,110 |
| **Specific indirect 1** | |  |  |  |  |  |  |
|  | **AUTMOT** |  |  |  |  |  |  |
|  | **SATFAM** |  |  |  |  |  |  |
|  | **FAMTYPE** | 0,078 | 0,016 | 4,974 | 0,000 | 0,049 | 0,110 |
| **Direct** |  |  |  |  |  |  |  |
|  | **AUTMOT** |  |  |  |  |  |  |
|  | **FAMTYPE** | 0,109 | 0,046 | 2,396 | 0,017 | 0,022 | 0,198 |
| **Effects from FAMTYPE to GPA** | | |  |  |  |  |  |
| **Sum of indirect** | | 0,040 | 0,008 | 4,983 | 0,000 | 0,025 | 0,056 |
| **Specific indirect 1** | |  |  |  |  |  |  |
|  | **GPA** |  |  |  |  |  |  |
|  | **AUTMOT** |  |  |  |  |  |  |
|  | **SATFAM** |  |  |  |  |  |  |
|  | **FAMTYPE** | 0,016 | 0,004 | 4,169 | 0,000 | 0,009 | 0,025 |
| **Specific indirect 2** | |  |  |  |  |  |  |
|  | **GPA** |  |  |  |  |  |  |
|  | **ACPERS** |  |  |  |  |  |  |
|  | **AUTMOT** |  |  |  |  |  |  |
|  | **SATFAM** |  |  |  |  |  |  |
|  | **FAMTYPE** | 0,003 | 0,001 | 3,080 | 0,002 | 0,002 | 0,006 |
| **Specific indirect 3** | |  |  |  |  |  |  |
|  | **GPA** |  |  |  |  |  |  |
|  | **ACPERS** |  |  |  |  |  |  |
|  | **HWAUTMOT** | |  |  |  |  |  |
|  | **SATFAM** |  |  |  |  |  |  |
|  | **FAMTYPE** | 0,005 | 0,001 | 3,160 | 0,002 | 0,002 | 0,008 |
| **Specific indirect 4** | |  |  |  |  |  |  |
|  | **GPA** |  |  |  |  |  |  |
|  | **ACPERS** |  |  |  |  |  |  |
|  | **SATFAM** |  |  |  |  |  |  |
|  | **FAMTYPE** | 0,006 | 0,002 | 3,198 | 0,001 | 0,003 | 0,011 |
| **Specific indirect 5** | |  |  |  |  |  |  |
|  | **GPA** |  |  |  |  |  |  |
|  | **HMPERS** |  |  |  |  |  |  |
|  | **SATFAM** |  |  |  |  |  |  |
|  | **FAMTYPE** | 0,005 | 0,002 | 2,935 | 0,003 | 0,002 | 0,009 |
| **Specific indirect 6** | |  |  |  |  |  |  |
|  | **GPA** |  |  |  |  |  |  |
|  | **HMPERS** |  |  |  |  |  |  |
|  | **AUTMOT** |  |  |  |  |  |  |
|  | **SATFAM** |  |  |  |  |  |  |
|  | **FAMTYPE** | 0,002 | 0,001 | 2,813 | 0,005 | 0,001 | 0,003 |
| **Specific indirect 7** | |  |  |  |  |  |  |
|  | **GPA** |  |  |  |  |  |  |
|  | **HMPERS** |  |  |  |  |  |  |
|  | **HWAUTMOT** | |  |  |  |  |  |
|  | **SATFAM** |  |  |  |  |  |  |
|  | **FAMTYPE** | 0,003 | 0,001 | 3,001 | 0,003 | 0,002 | 0,006 |
| **Effects from FAMTYPE to CHEATING** | | | |  |  |  |  |
| **Sum of indirect** | | –0,058 | 0,012 | –5,069 | 0,000 | –0,082 | –0,037 |
| **Specific indirect 1** | |  |  |  |  |  |  |
|  | **CHEATING** | |  |  |  |  |  |
|  | **AUTMOT** |  |  |  |  |  |  |
|  | **SATFAM** |  |  |  |  |  |  |
|  | **FAMTYPE** | –0,009 | 0,003 | –3,084 | 0,002 | –0,016 | –0,005 |
| **Specific indirect 2** | |  |  |  |  |  |  |
|  | **CHEATING** | |  |  |  |  |  |
|  | **HWAUTMOT** | |  |  |  |  |  |
|  | **SATFAM** |  |  |  |  |  |  |
|  | **FAMTYPE** | –0,009 | 0,003 | –2,971 | 0,003 | –0,016 | –0,004 |
| **Specific indirect 3** | |  |  |  |  |  |  |
|  | **CHEATING** | |  |  |  |  |  |
|  | **ACPERS** |  |  |  |  |  |  |
|  | **AUTMOT** |  |  |  |  |  |  |
|  | **SATFAM** |  |  |  |  |  |  |
|  | **FAMTYPE** | –0,003 | 0,001 | –3,157 | 0,002 | –0,006 | –0,002 |
| **Specific indirect 4** | |  |  |  |  |  |  |
|  | **CHEATING** | |  |  |  |  |  |
|  | **ACPERS** |  |  |  |  |  |  |
|  | **HWAUTMOT** | |  |  |  |  |  |
|  | **SATFAM** |  |  |  |  |  |  |
|  | **FAMTYPE** | –0,004 | 0,001 | –3,102 | 0,002 | –0,007 | –0,002 |
| **Specific indirect 5** | |  |  |  |  |  |  |
|  | **CHEATING** | |  |  |  |  |  |
|  | **ACPERS** |  |  |  |  |  |  |
|  | **SATFAM** |  |  |  |  |  |  |
|  | **FAMTYPE** | –0,006 | 0,002 | –3,120 | 0,002 | –0,010 | –0,003 |
| **Specific indirect 6** | |  |  |  |  |  |  |
|  | **CHEATING** | |  |  |  |  |  |
|  | **HMPERS** |  |  |  |  |  |  |
|  | **SATFAM** |  |  |  |  |  |  |
|  | **FAMTYPE** | –0,013 | 0,003 | –4,209 | 0,000 | –0,020 | –0,008 |
| **Specific indirect 7** | |  |  |  |  |  |  |
|  | **CHEATING** | |  |  |  |  |  |
|  | **HMPERS** |  |  |  |  |  |  |
|  | **AUTMOT** |  |  |  |  |  |  |
|  | **SATFAM** |  |  |  |  |  |  |
|  | **FAMTYPE** | –0,005 | 0,001 | –3,906 | 0,000 | –0,008 | –0,003 |
| **Specific indirect 8** | |  |  |  |  |  |  |
|  | **CHEATING** | |  |  |  |  |  |
|  | **HMPERS** |  |  |  |  |  |  |
|  | **HWAUTMOT** | |  |  |  |  |  |
|  | **SATFAM** |  |  |  |  |  |  |
|  | **FAMTYPE** | –0,009 | 0,002 | –4,364 | 0,000 | –0,014 | –0,006 |
